# Anti-Aging Effect of the Stromal Vascular Fraction/Adipose-Derived Stem Cells in a Mouse Model of Skin Aging Induced by UVB Irradiation

**DOI:** 10.3389/fsurg.2022.950967

**Published:** 2022-07-08

**Authors:** Jingru Wang, Yuanwen Chen, Jia He, Guiqiang Li, Xiaodong Chen, Hongwei Liu

**Affiliations:** ^1^ Department of Plastic Surgery, The First Affiliated Hospital of Jinan University, Guangzhou, China; ^2^ Department of Burn Surgery, First People’s Hospital of Foshan, Foshan, China; ^3^ Department of Burn and Plastic Surgery, The People's Hospital of Baoan shenzhen, Shenzhen, China

**Keywords:** adipose-derived stem cells, stromal vascular fraction, anti photo-aging, dose-effect relationship, cell-mediated therapy

## Abstract

Adipose-derived stem cells(ADSCs) have been used for anti-photo-aging. But the purification of ADSCs requires *in vitro* amplification and culture, there is considerable risk of direct treatment for patients. Stromal vascular fraction(SVF) is a biologically and clinically interesting heterogeneous cell population contains ADSCs. There are few reports on anti-aging effects of SVF in photo-aging skin. The present study investigated the anti-aging effect of stromal vascular fraction (SVF) and adipose-derived stem cells (ADSCs) injection in photo-aging skin. The relationship between the dosage of injection and effect was also discussed. Thirty healthy, 6-week-old, nude rats were randomly divided into the control and experimental groups. The experimental group needing ultraviolet B (UVB) irradiation five days per week, and a duration of 8 weeks. According to different dose regimens of SVF and ADSCs, experiment rats were randomly grouped as the model control group, low-dose (LD) treatment group, middle-dose (MD) treatment group and high-dose (HD) treatment group. At 7 and 28 days post-treatment, specimens were harvested for histological and immunohistochemical analysis. We found that certain concentrations of cells (MD and HD groups) could improve the texture of photoaged skin. Changes in the epidermal cell layer were clearly observed after 7 days of treatment. The epidermal layer becomes thinner and more tender. After 28 days of treatment, the dermal tissue was thickened and the collagen content and proportion were improved. All these indicators showed no significant difference between the same dosages in the two treatment groups. Our results demonstrate that SVF may have anti-aging potential in photo-aging skin and the ADSCs play an important role in SVF. SVF maybe a potential agent for photo-anging skin and the most effective dose of SVF was 10^6^ cells /100 µl/injection point. The proper injection interval may be 1.5 cm.

## Introduction

Since skin is the largest human organ, it's aging is the most intuitive form of aging. Skin aging is a complex biological process, which is influenced by endogenous (genetic, psychological, etc.) and exogenous factors (1). Under the combined action of these factors, the structure, function, and appearance of the skin changes.

Ultraviolet (UV) exposure is one of the major causative factors for skin aging. Ultraviolet B (UVB) radiation causes connective tissue damage more efficiently than longer wavelength radiation, so it is thought to play a major role in the production of skin wrinkles (2). Long-term UV exposure causes skin wrinkles, roughness, and loss of elasticity (3). Collagen bundles, as the main structural protein of human skin, maintain the skin tensity. Collagen type I (85%–95%) and collagen type III (10%–15%) are the main protein components of the dermis in adults (2, 4). Long-term sun exposure hinders the synthesis of collagen I, ultimately leading to a reduction in the mature collagen bundles and increase in the immature collagen type III, which causes skin wrinkles and sagging (3, 5). UV irradiation can also stimulate dermal inflammatory reaction and activate the inflammatory mediators and cytokines to release resolvase, which gradually dissolves the contents of the dermis (6). Continuous or repetitive protease hydrolysis of extracellular matrix (ECM) is one of the basic characteristics of skin aging process. The imbalance between synthesis and degradation of dermal ECM also leads to wrinkles. The synergistic effect of matrix metalloproteinases (MMPs) family majorly contributes to the degradation of ECM (3, 6, 7) especially MMP-1 and MMP-3. In addition to the changes in the dermis, the histological changes, and the changes in the epidermis due to photo-aging include hyperkeratosis, irregular thickening or atrophy and abnormal proliferation of basal layer cells (6).

Cell therapy has become a primary treatment in all fields of modern medicine. Stem cells have been used for wound healing and tumor treatment. Adipose-derived stem cells (ADSCs) are pluripotent mesenchymal stem cells, which are isolated from adipose tissue by liposuction (8). Numerous studies have shown that ADSCs stimulate the proliferation, secretion, and migration of fibroblast cells, and promote blood vessel regeneration by their paracrine and autocrine effects during the repair and reconstruction of skin tissue injury (8–12). Moreover, previous studies have suggested that ADSCs improve the quality of aging skin by promoting collagen synthesis and anti-oxidation (13, 14). However, the purification of ADSCs requires *in vitro* amplification and culture. There is considerable risk of direct treatment for patients. Stromal vascular fraction (SVF) is a biologically and clinically interesting heterogeneous cell population, which can be employed directly or cultured for selection and expansion of an adherent population, called the ADSCs, whose content is 20%–30% (15). There are very few reports on anti-aging effects of SVF in photo-damaged aged skin. Thus, in the present study, we employed a UVB-induced nude rat model and subcutaneously injected SVF to explore its influence on photo-aging skin. We also explored the action of ADSCs using the corresponding dose relationship to SVF.

## Materials and Methods

### Isolation of SVF

Subcutaneous adipose tissue was obtained by tumescent liposuction from healthy women donors (*n* = 6, aged 28–34 years) after informed consent, as approved by the institutional review boards. The adipose tissue samples were washed three times with phosphate buffered saline (PBS), and then the washed aspirates were digested with 0.1% collagenase type I (Sigma, America) for 30 min at 37°C with gentle constant shaking, and centrifuged at 300 g for 10 min. Pellets were resuspended in PBS and filtered with 200 µm nylon mesh followed by centrifugation at 300 g for 10 min. Then, the pellets were resuspended in lysis buffer (0.3% NaCl solution) for 10 min at 37°C, and centrifuged for 5 min at 300 × g to obtain the SVF (16). The percentage of ADSCs in SVF was examined by surface marker expression using flow cytometric analysis with an LSR2 (Becton Dickinson, San Jose, CA). The following monoclonal antibodies were used: CD44 (Miltenyi Biotec, Germany), CD45 (Miltenyi Biotec, Germany), CD34 (BD Biosciences, San Diego, CA), CD29 (BD Biosciences, San Diego, CA), HLA-DR (BD Biosciences, San Diego, CA).

### Isolation and Culture of ADSCs

**Figure 1:**
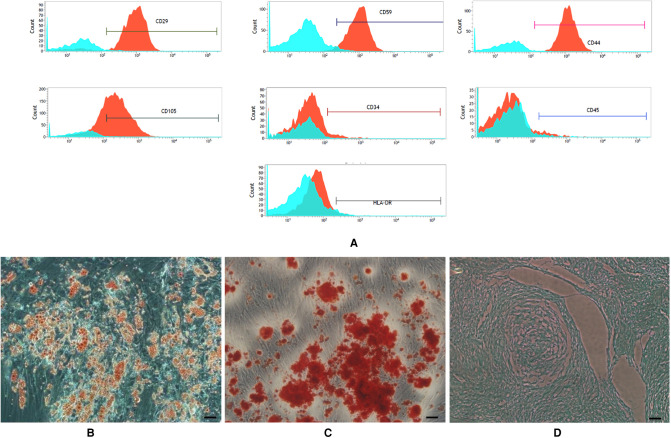
(**A**) expression of surface marker of the third generation of ADSCs. Flow cytometry labeling of surface markers showed (**B–D**): Multi-lineage differentiation potential of adipose-derived stem cells (ADSCs). (**B**) Oil Red O staining. (**C**) Alizarin red staining. (**D**) Alcian blue staining (scale bars = 50 µm).

SVF was isolated as mentioned above and then the cell number of SVF was counted with a cell counter. For culture of ADSCs, the pellet was resuspended in DMEM/low glucose with 10% fetal calf serum, supplemented with 100 U/ml of penicillin and 100 µg of streptomycin, and transferred to T25 flasks at a density of 10^4^ nucleated cells. Primary cells were cultured for three days, non-adherent cells were removed by replacing the medium and the adherent cells were cultured for another day, defined as “Passage 0” (P0). The medium was changed every three days until reaching 70%–80% confluence, the adherent cells were detached by trypsin containing EDTA (Gibco USA) and plated in the same medium at a density of 2,000 cells/cm^2^. *In vitro* cultured ADSCs were subjected to adipogenic differentiation, osteogenic differentiation and chondrogenic differentiation, and detected using Oil-red O, Alizarin red, and Alcian blue staining (Figure 1). Cells of P3 were used in the subsequent experiments.

### UVB-induced Aging Model

Thirty female nude rats were purchased from Charles River Laboratories [SCXK (Jing) 202012-0001]. All rats were housed in climate-controlled quarters (22 ± 1°C, 40%–60% humidity) at the institute of laboratory animal science, Jinan university, with a 12/12 h light/dark cycle. They were acclimatized for one week prior to study initiation, and allowed free access to food and water. The rats were randomly divided into the control group (*n* = 6) and the experimental group (*n* = 24). The experimental model group was further randomly divided into four groups (*n* = 6 each).

The skin wrinkles were induced by UVB irradiation as previously described by Kim Bissett et al (17). The rats were irradiated dorsally using the UVB lamps (Toshiba, 15, W 2 tube, irradiance 290–320 nm) for eight weeks, five times a week. The distance between the lamps and the animals' backs was 50 cm. During exposure, the animals could freely move around in the cages. Irradiation intensity at the skin surface was measured using a UV meter. The irradiation dose was one minimal erythemal dose (MED; 60 mJ/cm^2^) in the first week, two MED in the second week, three MED in the third week, and four MED in the fourth week, through eight weeks. The total UVB dose was approximately 130 MED (7.8 J/cm^2^).

### Measurement of the Injection Area

100 µl methylene blue solution was subcutaneously injected into the rats. The diffusion range was measured by a ruler.

### Animal Experiments

After wrinkle induction, the nude rats of the UVB model group were randomly divided into four groups (*n* = 6). Three groups of animals received cell therapy. SVF (1 × 10^4^, 1 × 10^5^, 1 × 10^6^ cells/100 µl/site) were subcutaneously injected into the restricted area of one side of dorsal skin, and ADSCs (2 × 10^3^, 2 × 10^4^, 2 × 10^5^ cells/100 µl/site) were subcutaneously injected into the other side (Our experiments confirm that the average percentage of ADSCs in SVF isolated from adipose tissue of healthy women was 25.79 ± 5.7%. To facilitate counting and confirm the minimum effective quantities we chose 20% as the experimental parameters.) The same volume of physiological saline was injected into the UVB model control group and the control group. We marked the injection point daily with a marking pen. One week after injection, biopsy specimens were taken with a 5-mm punch. Skin samples were fixed with 4% paraformaldehyde for histopathology. Four weeks after injection, all animals were sacrificed and skin samples were collected. The skin tissue was divided into two parts. One part was fixed with 4% paraformaldehyde as aforementioned and the other part was stored in liquid nitrogen for subsequent analysis.

### Histological Examination

Skin tissues were dehydrated and paraffin-embedded after 24 h fixation, and serially sectioned (4 µm) for hematoxylin and eosin (H&E) staining. The pictures of each section were taken under ×40 or ×400 magnification. Dermal thickness (the distance between subcutaneous fat and the dermoepidermal junction) and epidermal thickness (the distance from the granular layer to basal layer) were measured randomly at five sites on each picture.

### Quantitative Polymerase Chain Reaction (qPCR) Analysis

Total RNA was extracted from skin samples stored in liquid nitrogen using TRIzol (Invitrogen, Carlsbad, CA, USA), and then resuspended in RNase-free water. The RNA was electrophoresed in 1% agarose gels to confirm its quality and quantity. Equal amounts of RNA (1 µg) were reverse-transcribed using a cDNA synthesis kit (Toyobo CO. LTD., Japan).

qPCR was performed on a CFX 96™ Real-Time PCR detection system (Bio-Rad mini option, USA) using SYBR Green I PCR Master Mix (Takara BIOINC., Japan). The following oligonucleotides were used as primers: the internal control *β*-actin (F:5′-GAGTACAACCTTCTTGCAGCTC-3′ and R: 5′-CATACCCACCATCACACCCTG-3′), rat collagen I-α_1_ (F: 5′-GATGGACTCAACGGTCTCCC-3′ and R: 5′-CGGCCACCATCTTGAGACTT-3′), rat collagen III (F: 5′-CTGAAGGGCAGGGAACAACT-3′, R: 5′-ATCCCGAGTCGCAGACACATA-3′), and rat MMP-3 (F: 5′-GATGGACTCAACGGTCTCCC-3′, R: 5′-CGGCCACCATCTTGAGACTT-3′). The two-step qPCR amplification standard procedure was as follows: Step 1, 95°C, 30 s; Step 2, 40 cycles with 95°C, 5 s and 60°C, 30 s. The quantity of PCR products was calculated from the cycle threshold value. The levels of gene expression were normalized with those of *β-actin* gene. The data was calculated with the formula of relative expression quantity = 2^−△△Ct^.

### Immunohistochemistry Staining of Ki67

Immunohistochemistry was used to detect the cell proliferation of basal cell layer. Two types of markers were used, respectively: Anti-Ki67 antibody (Abcam, Cambridge, UK). Paraffin sections (4 µm) of each group of 7-day skin samples were dehydrated and rehydrated before immunohistochemical staining. After rehydration, the sections were incubated for 5 min in PBS, followed by microwave antigen retrieval for 10 min in sodium citrate buffer (pH = 6.0). The slide was removed from the microwave oven and allowed to cool at room temperature. Slides were washed with PBS for 5 min, three times and incubated in 3% H_2_O_2_ for 20 min, washed again in PBS, and blocked using a protein block solution for 30 min at room temperature. Then, sections were incubated with the primary antibody at 4°C overnight (over 10 h). The next day, the sections were removed from the fridge and rewarmed to room temperature for 1 h. The sections were washed with PBS for three times and then incubated with a biotinylated secondary antibody for 1 h. Slides were counterstained with diaminobenzidine and visualized on an Olympus microscope. The numbers of Ki67-positive nuclei were quantified using the Image J software on five non-consecutive tissue sections for each image, respectively.

### Statistical Analysis

All values are expressed as mean ± standard deviation. The data were evaluated by analysis of variance followed by a Newman-Keuls' test for multiple comparisons. The difference was considered to be significant if *P* < 0.05.

## Results

### Percentage of ADSCs in SVF

Flow cytometric analyses showed that the average percentage of ADSCs in SVF isolated from adipose tissue of healthy women was 25.79 ± 5.7%.

### Characterization of ADSCs

ADSCs expanded easily *in vitro* and exhibited a fibroblast-like morphology. Characteristic expressions of stem cell-related surface markers were confirmed by flow cytometry. ADSCs expressed CD29, CD59, CD44 and CD105, but not CD34, CD45 and HLA-DR (Figure 1A). ADSCs were incubated in media known to induce an adipogenic, osteogenic, or chondrogenic lineage. Adipogenic, osteogenic, and chondrogenic differentiation was confirmed by Oil Red O staining (Figure 1B), Alizarin red S staining (Figure 1C), and Alcian blue staining (Figure 1D).

### Diffusion Range of Subcutaneously Injected Methylene Blue Solution in the Back Skin of Rats

**Figure 2:**
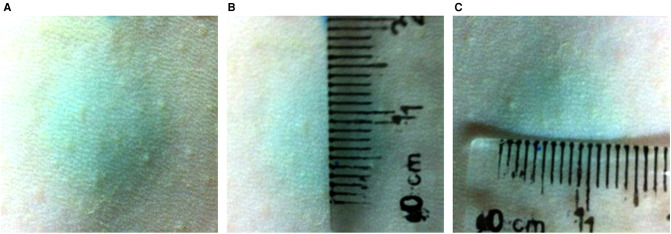
The diffusion range of 100 µl methylene blue solution subcutaneous injection the back skin of rats. The methylene blue solution diffused as a circle with a diameter of 1.5 cm after subcutaneous injection.

The methylene blue solution diffused as a circle with a diameter of 1.5 cm after subcutaneous injection (Figure 2).

### Effect of SVF and ADSCs Injection on Epidermal Thickness and the Proportion of Stratum Corneum in the Epidermis

**Figure 3:**
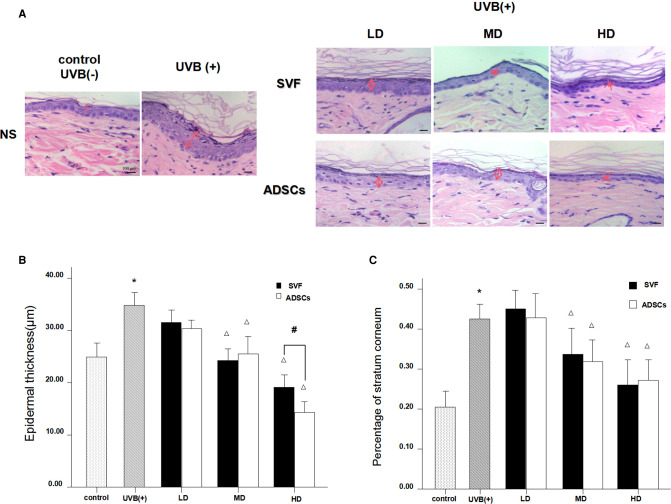
Haematoxylin staining showed the changes of epidermal thickness of nude rats exposed to UVB 7 days after SVF treatment and ADSCs treatment (×400). (**A**) UVB exposure decreased the epidermal thickness of nude rats and SVF and ADSCs treatment increased the epidermal thickness of aging skin. (**B**) The epidermal thickness of UVB exposed group and SVF and ADSCs treatment groups. (**C**) Percentage of stratum corneum of UVB exposed group and SVF and ADSCs treatment groups. Mean ± SD. *n* = 6. *P < 0.05 vs control group. ^△^*P* < 0.05 vs UVB(+) group. ^#^*P* < 0.05 between the groups with line marker.

H&E staining showed significant changes in skin samples from treated rats compared to control rats after seven days (Figure 3A). Measurement of the epidermal thickness showed decrease in the MD and HD groups of SVF and ADSCs (*P* < 0.05) (Figure 3B). No difference was observed in the matched groups between SVF and ADSCs (Figure 3B). The proportion of stratum corneum in the epidermis was also decreased in the MD and HD groups of SVF and ADSCs (*P* < 0.05) (Figure 3C). No difference was observed in the matched groups between SVF and ADSCs (Figure 3C).

### Analysis of the Proliferation of the Basal Layer of Epidermis

**Figure 4:**
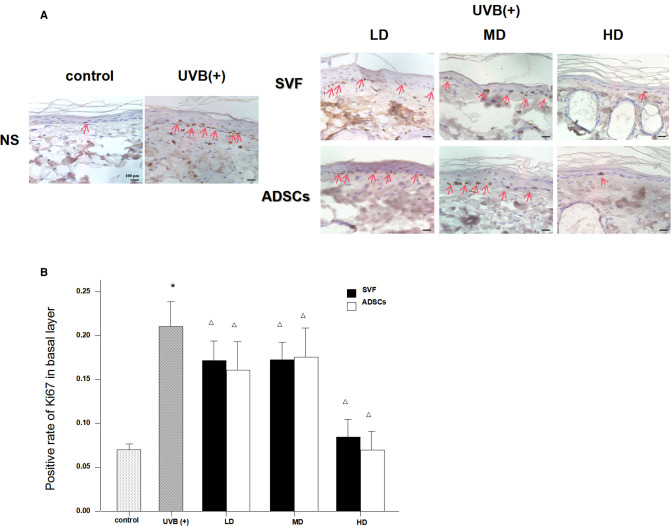
Ki67 immunostain showed the proliferation of basal cell nuclei that treated with ADSCs and SVF after 7 days (×400). (**A**) UVB exposure stimulated abnormal proliferation of the basal layer cells. SVF and ADSCs treatment decreased the abnormal proliferation. (**B**) All of the treated groups had lower proliferation rates (*p* < 0.05). The efficacy was significantly higher in the HD treatment group. No difference was observed in the matched groups between SVF and ADSCs. Mean ± SD. *n* = 6. *P < 0.05 vs control group. △*P* < 0.05 vs UVB (+) group.

UVB exposure stimulated abnormal proliferation of the basal layer cells. SVF and ADSCs treatment decreased the abnormal proliferation (Figure 4A). We measured Ki67-positive nuclei to further confirm the efficacy. As shown in Figure 4B all of the treated groups had lower proliferation rates (*P* < 0.05). The efficacy was significantly higher in the HD treatment group. No difference was observed in the matched groups between SVF and ADSCs (Figure 4B).

### Effect of SVF and ADSCs Injection on Dermal Thickness

**Figure 5:**
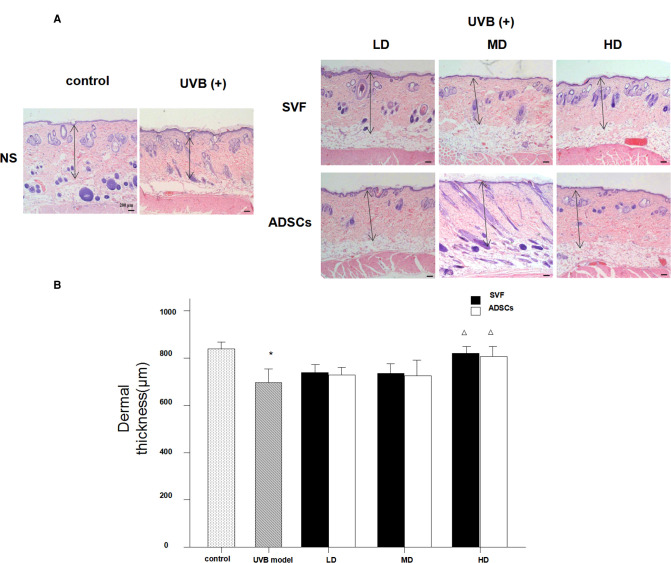
Haematoxylin staining showed the changes of dermal thickness of nude rats exposed to UVB 28 days after SVF treatment (×40). (**A**) UVB exposure decreased dermal thickness and SVF and ADSCs treatment increased dermal thickness of aging skin. (**B**) The dermal thickness of UVB exposed group and SVF and ADSCs treatment groups. Mean ± SD. *n* = 6. *P < 0.05 vs control group. ^△^*P* < 0.05 vs UVB model group.

Figure 5A shows the histological measurements of the dermal thickness of the nude rats by H&E staining after 28 d. The dermal thickness increased in the HD groups with SVF and ADSCs treatment (*P* < 0.05) (Figure 5B). No difference was observed in the matched groups between SVF and ADSCs (Figure 5B).

### Relative mRNA Expression of Collagen Type I, Collagen Type III and Matrix Metalloproteinase 3 (MMP-3) in Dermis

**Figure 6:**
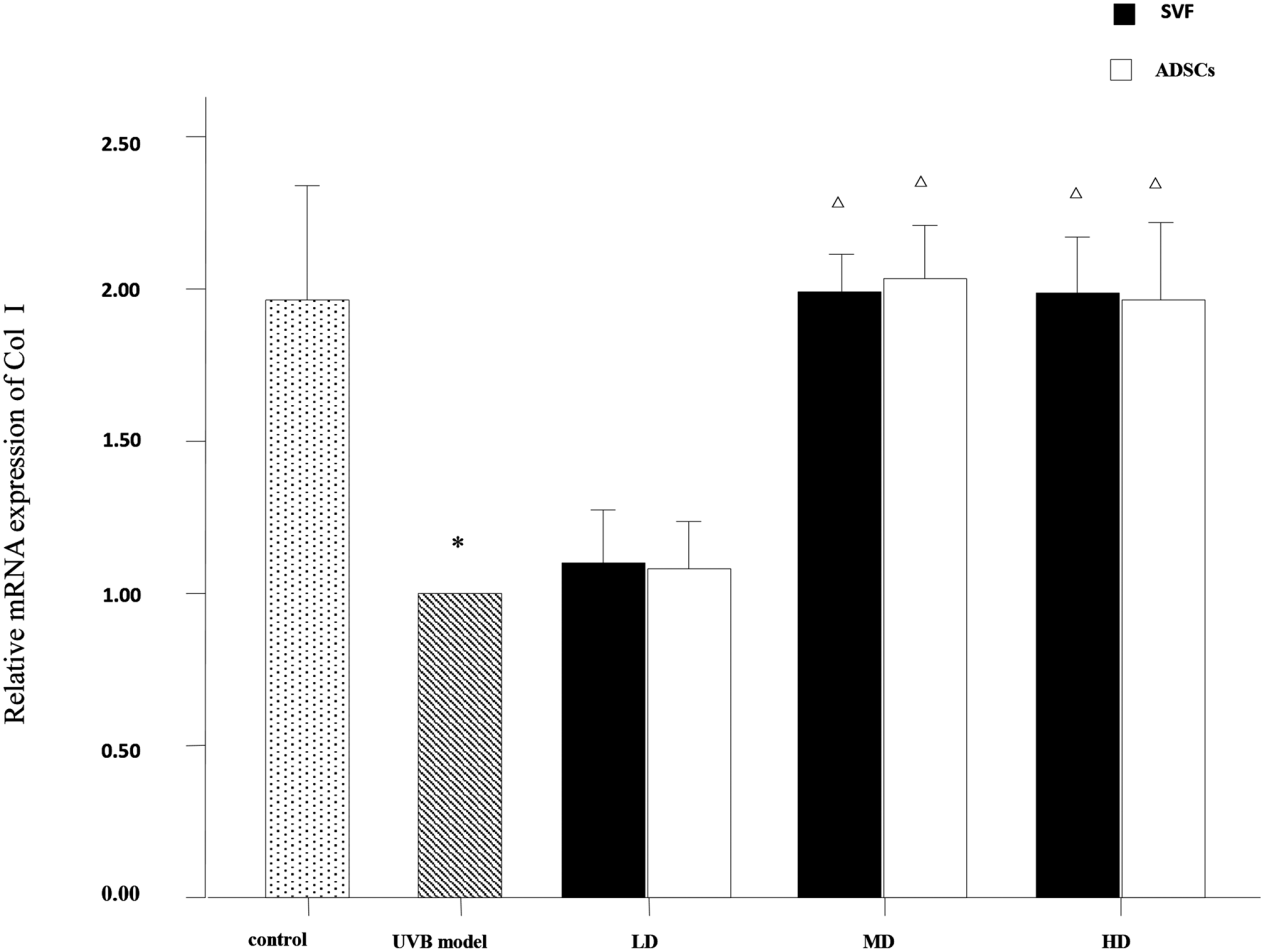
Quantitative polymerase chain reaction (qPCR) showed the relative mRNA expression of collagen I of nude rats exposed to UVB 28 days after SVF and ADSCs treatment. Mean ± SD. *n* = 6. *P < 0.05 vs control group. ^△^*P* < 0.05 vs UVB model group.

**Figure 7:**
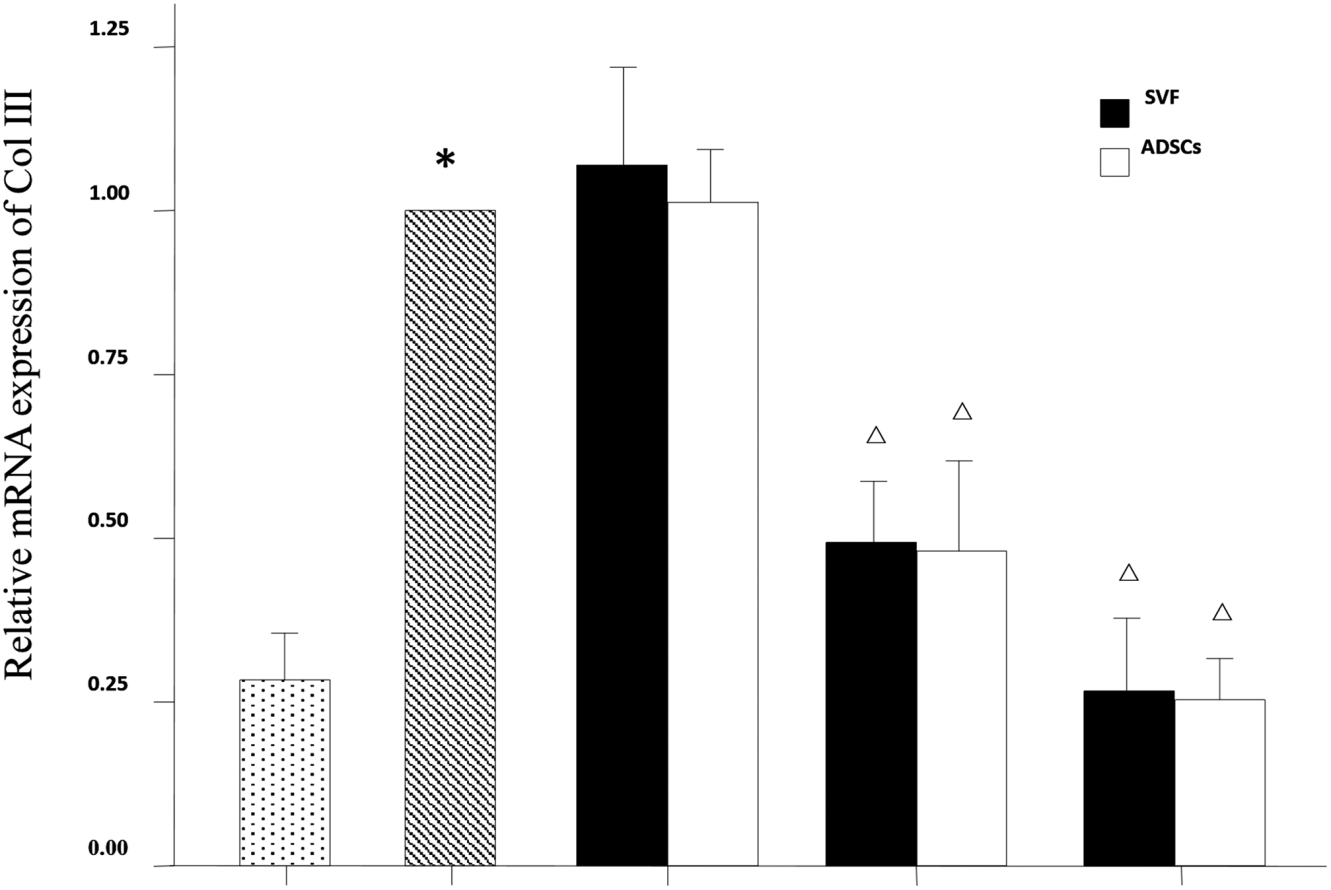
qPCR showed the relative mRNA expression of collagen III of nude rats exposed to UVB 28 days after SVF and ADSCs treatment. Mean ± SD. *n* = 6. *P < 0.05 vs control group. ^△^*P* < 0.05 vs UVB model group.

**Figure 8:**
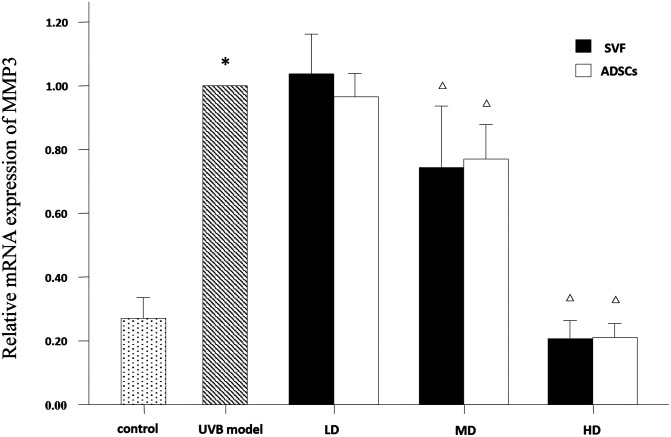
qPCRed the relative mRNA expression of MMP3 of nude rats exposed to UVB 28 days after SVF and ADSCs treatment. Mean ± SD. *n* = 6. *P < 0.05 vs control group. ^△^*P* < 0.05 vs UVB model group.

The mRNA expression levels of collagen type I, collagen type III and MMP-3 were compared between the experimental groups. As shown in Figure 6, the relative mRNA expression of collagen type I was increased in the MD and HD groups of SVF and ADSCs (*P* < 0.05). As shown in Figure 7, collagen type III expression was decreased in the MD and HD groups of SVF and ADSCs (*P* < 0.05). The relative mRNA expression of MMP-3 was decreased in all groups (Figure 8), and the MD and HD groups showed statistically significant decrease (*P* < 0.05). No difference was observed in the matched groups between SVF and ADSCs in the above targets (Figures 6–8).

## Discussion

Cell therapy has become an important component in the development of modern medicine. For example, stem cells, which have reproductive capacity, are used to treat therioma or restore organ functions after damage by disease or trauma. Embryonic stem cells obtained from fetuses were considered to be the best source, and have been thoroughly investigated. However, there are serious ethical issues and carcinogenic potential that hinder their clinical application (18). Therefore, adult stem cells have been used as they do not have the ethical problems related to embryonic stem cells. In recent years, literature related to SVF cells and ADSCs has been considerably augmented. These studies have demonstrated the safety and efficacy of autologous SVF use in regenerative cell therapy for wound healing, skeletal regeneration, cardiovascular and peripheral vascular diseases, and tissue engineering (19, 20). However, there are few reports on its anti photo-aging potential. SVFs are widely present in fat tissue, which contains 20%–30% ADSCs, and can be easily harvested during aesthetic lipoaspiration procedures. Hence, sufficient quantity of cells can be easily obtained from patients with less pain. The cells can be immediately used during the surgery. Moreover, it can avoid the biological safety issues and reduce the risk of infection due to absence of exogenous substance during the operation. The present study demonstrated that SVF, which contains ADSCs, has effective anti-wrinkle properties, as observed by the effects on epidermal thickness, epidermal stratum corneum thickness, abnormal proliferation of basal layer, dermal thickness and relative mRNA expressions of dermal collagen I, collagen III and MMP-3.

The epidermal turnover time of healthy human skin is 28 days. However, in the case of foreign invasion and destruction of the stratum corneum, the basal cell division is activated to remove foreign bodies for repair. The chemical (organic solvents, etc.) or physical (UVB, etc.) irritation may trigger epidermal hyperplasia and abnormal dyskeratosis (21). In our study, we observed that long-term UVB irradiation increased the epidermal thickness, especially in the stratum corneum, and the proportion of stratum corneum was obviously increased in the epidermis. Moreover, long-term exposure to UV irradiation led to disarrangement of the basal cells and changes in the morphology of nuclei. UV irradiation activated the proliferation of epidermal keratinocytes, which is an adaptive process of cell apoptosis, and DNA damage can cause abnormal proliferation of cells in the absence of repair (22, 23). After the SVF and ADSCs treatment, the abnormal proliferation of basal layer cells was significantly decreased. The proliferating phase cells were similar to normal skin when the cell concentration reached 10^6^/100 µl in the SVF treatment group. The same result was observed at the cell concentration of 2 × 10^5^/100 µl in the ADSCs treatment group. Hence, SVF was involved in the regulation of anti-UV damage in photo-aging skin, and ADSCs played a major role in this process. The underlying mechanism needs further study. SVF possibly repairs the epidermal damage of UV irradiation by the following processes: (1) SVF can inhibit the abnormal proliferation of basal cells; (2) SVF can accelerate the epidermal turnover rate; (3) SVF facilitates the division, maturity and loss of skin cells in a renewed state of equilibrium.

Photo-aging is a complex reparative process, with similarities to pathological skin wounds. It has been reported that adult stem cells can promote the proliferation of fibroblasts and the formation of new blood vessels by secreting cytokines, and promote the growth and epithelialization of granulation tissue, in order to promote wound healing (9, 10); while collagen repair is facilitated by promoting the secretion of fibroblasts in dermal tissue (11, 13, 15). Collagen accounts for about 90% of the human dermal protein and the major collagenous constituents of healthy dermis are the collagen type I (85%–95%) and type III (10%–15%) (2, 4). Aging decreases the content of collagen I and increases collagen III ratio in the same part of the skin, which is more pronounced in the UVB-exposed area (24). The decrease in collagen content and the change in collagen ratio leads to skin relaxation and eventual collapse to form the wrinkles (1, 23). These changes have been shown to correlate well with clinical severity of photo-aging (4). The present study showed that subcutaneous injection of SVF can increase the thickness of dermis, promote the relative mRNA expression of collagen type I, and inhibit the relative mRNA expression of collagen type III. Both middle-dose and high-dose of SVF treatment could improve the proportion of collagen type I and type III in dermis, which resembled young and healthy skin tissue.

Collagen reduction results from a combination of increased enzymatic breakdown *via* the actions of MMPs (5). MMPs are a family of proteins containing zinc that can cause tissue damage by degradation of the ECM leading to wrinkle formation (4). Excessive UV irradiation enhances the secretion of MMPs by keratinocytes, fibroblasts, and inflammatory cells. MMP-1, MMP-3 and MMP-9 are stimulated by UV irradiation, and alter collagen type I, which is related to the quality of the skin. MMP-1 initiates collagen fiber fracture process and MMP-3 leads to its degradation (5). MMP-3 can also activate pro-MMP-1. The amount of MMP-3 in the dermal tissue has a direct impact on the degradation efficiency of collagen (4, 11). Therefore, MMP-3 expression is an important determinant of wrinkle formation. MMP-3 mRNA expression can be used as an indicator of altered collagen content in the skin (11). The results of the present study showed that the relative expression of MMP-3 mRNA was decreased in all the SVF treatment groups. We hypothesized that SVF may hinder the degradation rate of collagen protein by down-regulating the expression of MMP-3 mRNA, which leads to a new homeostasis between synthesis and degradation of collagen protein and increases the collagen content in order to improve the quality of photo-aging skin.

The SVF contains multiple components, it is not clear whether the efficacy of anti-aging is attributable to the ADSCs or other components of fat tissue. The results of this study showed no significant difference in the corresponding dose group between SVF and ADSCs in all observed targets. Therefor, we infer that ADSCs is the main cellular component of SVF, which has a major effect in increasing the dermal thickness and collagen content (1, 18).

The therapeutic role of SVF in photo-aging has been demonstrated, but how to put it into clinical application is still a question worth exploring. The use dose and injection method of SVF were also discussed in our study. Based on the operability and clinical application, we selected low, medium and high doses (1 × 10^4^, 1 × 10^5^, 1 × 10^6^cells/100 µl/site) of SVF. We found that it acted on the epidermis and germinal layer cells when the cell numbers reached 10^5^cells/100 µl/site. But this treatment concentration did not improve the dermal thickness. Quantitative polymerase chain reaction (qPCR) analysis show that the relative mRNA expression of collagen type I was increased and collagen type III expression was decreased in MD group. Due to the deficiency of our experiments, the protein content in the dermis was not further determined, so we cannot deny that this is an effective concentration. Therefore, we considered that 10^5^cells/100 µl/site as the effective concentration, but 10^6^cells/100 µl/site as the optimal concentration. To determine the mode of injection, 100 µl methylene blue solution was subcutaneously injected into the rats. The methylene blue solution diffused as a circle with a diameter of 1.5 cm after subcutaneous injection. This suggests that we should be 1.5 cm apart between the two injection points.

## Conclusions

In summary, our study demonstrated that SVF had anti photo-aging effect in the UVB-irradiated mouse model. By comparing the effect of SVF and corresponding dose of ADSCs, ADSCs were found to play a major role in SVF. Although the multiplication capacity of basal cells and the relative expression of MMP-3 mRNA were significantly improved at all doses of SVF, analyses of epidermal and dermal thicknesses and the relative mRNA expression of collagen type I and type III suggested that the clinical dose of SVF in humans should be the high-dose in mice, which is 10^6^cells/100 µl/site. The methylene blue solution injection in rat skin indicated that the best injection interval is 1.5 cm. Although some progress has been made on the use of SVF, the underlying mechanism and the role of other endogenous cells need to be addressed.

## Data Availability

The original contributions presented in the study are included in the article/Supplementary Material, further inquiries can be directed to the corresponding author/s.
